# Mutation of YL Results in a Yellow Leaf with Chloroplast RNA Editing Defect in Soybean

**DOI:** 10.3390/ijms21124275

**Published:** 2020-06-16

**Authors:** Xiaowei Zhu, Yi Pan, Zhi Liu, Yucheng Liu, Deyi Zhong, Zongbiao Duan, Zhixi Tian, Baoge Zhu, Guoan Zhou

**Affiliations:** 1 State Key Laboratory of Plant Cell and Chromosome Engineering, Institute of Genetics and Developmental Biology, Innovative Academy of Seed Design, Chinese Academy of Sciences, Beijing 100101, China; xwzhu@saas.sh.cn (X.Z.); ypan@genetics.ac.cn (Y.P.); zliu@genetics.ac.cn (Z.L.); ychliu@genetics.ac.cn (Y.L.); zhongdeyi@caas.cn (D.Z.); zbduan@genetics.ac.cn (Z.D.); zxtian@genetics.ac.cn (Z.T.); 2 Horticulture Research Institute, Shanghai Academy of Agricultural Sciences, Shanghai 201403, China; 3 Agricultural Genomics Institute, Chinese Academy of Agricultural Sciences, Shenzhen 518120, China; 4 College of Advanced Agricultural Sciences, University of Chinese Academy of Sciences, Beijing 100049, China

**Keywords:** soybean, *yellow leaf* mutant, photosynthesis, chloroplast RNA editing

## Abstract

RNA editing plays a key role in organelle gene expression. Little is known about how RNA editing factors influence soybean plant development. Here, we report the isolation and characterization of a soybean *yl* (*yellow leaf*) mutant. The *yl* plants showed decreased chlorophyll accumulation, lower PS II activity, an impaired net photosynthesis rate, and an altered chloroplast ultrastructure. Fine mapping of *YL* uncovered a point mutation in *Glyma.20G187000*, which encodes a chloroplast-localized protein homologous to *Arabidopsis thaliana* (*Arabidopsis*) ORRM1. *YL* is mainly expressed in trifoliate leaves, and its deficiency affects the editing of multiple chloroplast RNA sites, leading to inferior photosynthesis in soybean. Taken together, these results demonstrate the importance of the soybean YL protein in chloroplast RNA editing and photosynthesis.

## 1. Introduction

Photosynthesis is a vital process in which plants convert light energy into chemical energy [1,2]. In higher plants, chlorophyll *a* and *b* are the two major pigments located in the thylakoid membrane of photosynthetic organisms [2]. These chlorophyll molecules play essential roles in harvesting light energy and transferring that energy to reaction centers of the photosystem [2,3]. In the past few decades, tremendous research has been conducted on chlorophyll-deficient mutants in model plants, but the molecular genetic mechanisms underlying these soybean chlorophyll-deficiency mutations are not well understood.

For soybean, 15 chlorophyll-deficient mutant genes were reported to be mapped to chromosomes [4]. Of these genes, six lethal yellow mutant genes, including *Y11*, *Y18/Y18_1*, *Y18_2*, *YL_PR350*, *PsbP*, and *CD-5*, were mapped to chromosomes 13, 14, 17, 15, 3 and 15, respectively [5,6,7,8,9]. The other nine viable yellow mutant genes, including *Y9*, *Y10*, *Y12*, *Y13*, *Y17*, *Y20*, *Y23*, *Tic110*, and *Cd1*, were mapped to chromosomes 15, 3, 6, 13, 15, 12, 13, 2, and 10, respectively [8,10,11,12,13,14,15,16]. However, only the function of *Y11* and *Y9* genes in yellow foliage was validated by complementary analysis [6,9].

RNA editing is a post-transcriptional modification process that changes the sequence of RNA molecules so that the information in the mature RNA differs from that defined in the genome [17]. In land plants, RNA editing occurs in transcripts of chloroplasts and mitochondria. There are 20–60 chloroplasts and over 300 RNA mitochondrial editing sites in most flowering plants [17,18]. RNA editing is performed by an editosome that is assembled via protein-protein/RNA interactions [19]. Several organelle RNA recognition motif-containing (ORRM) proteins are known to be essential RNA editing factors. ORRM1 controls 62% of chloroplast editing sites in *Arabidopsis* and 81% of editing sites in maize, with the *Zm-orrm1* mutant exhibiting a pale green phenotype [20]. ORRM6 is primarily required for editing *psbF*-C77 and *accD*-C794 sites in *Arabidopsis* chloroplasts [21]. In addition, ORRM2, ORRM3, ORRM4 and ORRM5 are mitochondrial RNA editing factors [22,23,24]. Moreover, pentatricopeptide repeat (PPR) proteins, multiple organellar RNA editing factor (MORF)/RNA editing factor interacting proteins (RIPs), organelle zinc finger 1 (OZ1), protoporphyrinogen oxidase 1 (PPO1), and genomes uncoupled 1 (GUN1) have been identified as components of the plant RNA editosome [19,25,26,27,28,29,30].

Here, we report the characterization of a soybean *yellow leaf* (*yl*) mutant with chlorophyll deficiency and impaired photosynthesis. Fine mapping and DNA sequencing showed that *YL* encodes a GmORRM1. We showed that multiple chloroplast RNA editing sites were changed in *yl* mutants. Our findings provide new insight into the function of YL in soybean photosynthesis.

## 2. Results

### 2.1. yl Plants Show Yellow Leaves and Abnormal Photosynthesis at the Seedling Stage

**Figure 1:**
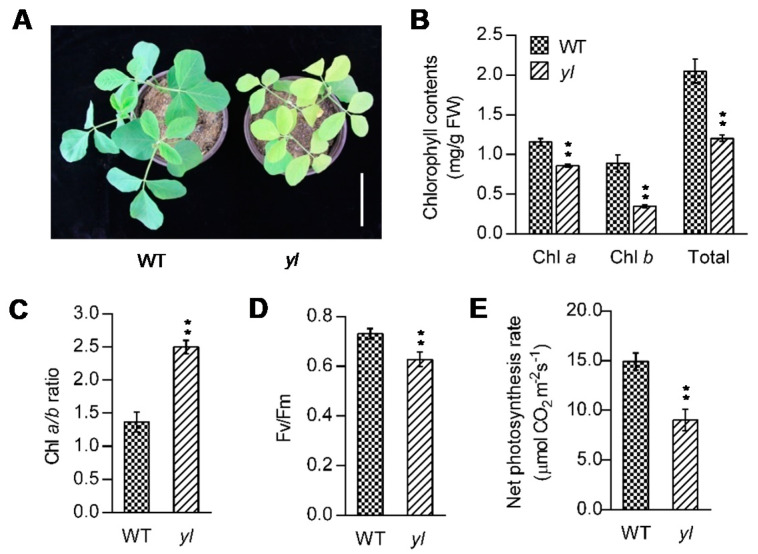
Phenotypic characterization of the wild type and *yl* mutant plants. (**A**) Two-week-old wild type (left) and *yl* (right) seedlings. (**B**) Chlorophyll contents of the third leaf of the wild type (left) and *yl* (right) plants. (**C**) The Chl *a*/*b* ratio of the wild type (left) and *yl* (right) leaves. (**D**) The maximum photochemical efficiency of PSII measured by the Fv/Fm chlorophyll fluorescence ratio in the wild type (left) and *yl* (right) leaves. (**E**) The net photosynthesis rate of the wild type (left) and *yl* (right) plants. Error bars (**B**–**E**) represent the mean ± SD (*n* ≥ 3); ** indicates a significant difference at the 0.01 level. The *p* values were calculated by Student’s t-test. Bar = 5 cm in (**A**).

The *yl* mutant was identified from the soybean cultivar Jindou 23 mutagenized with ethyl methane sulfonate (EMS). The *yl* mutant plants exhibited conspicuous yellow leaves (Figure 1A). Chlorophyll (Chl) analysis revealed that the Chl *a*, *b* and total Chl contents of wild type (Jindou 23) leaves were 1.15 mg/g, 0.89 mg/g and 2.04 mg/g, respectively (Figure 1B). In contrast, the Chl *a*, *b* and total Chl contents of *yl* leaves were 0.86 mg/g, 0.34 mg/g and 1.20 mg/g, respectively (Figure 1B). In addition, the Chl *a*/*b* ratio of *yl* leaves was significantly increased compared to that of the wild type (Figure 1C). To evaluate whether the photosynthesis of *yl* leaves was affected, we measured the maximum photochemical efficiency of photosystem II (PSII), defined as the Fv/Fm and net photosynthesis rate. The *yl* leaves displayed obviously decreased Fv/Fm values (wild type, 0.73; *yl*, 0.62) (Figure 1D). Moreover, the net photosynthesis rate of *yl* leaves was 9.03 μmol CO_2_ m^−2^s^−1^, which was only 60% that of wild type leaves (14.93 μmol CO_2_ m^−2^s^−1^) (Figure 1E). Therefore, the mutation in *yl* causes not only a reduction in chlorophyll contents but also abnormalities in photosynthesis.

**Figure 2:**
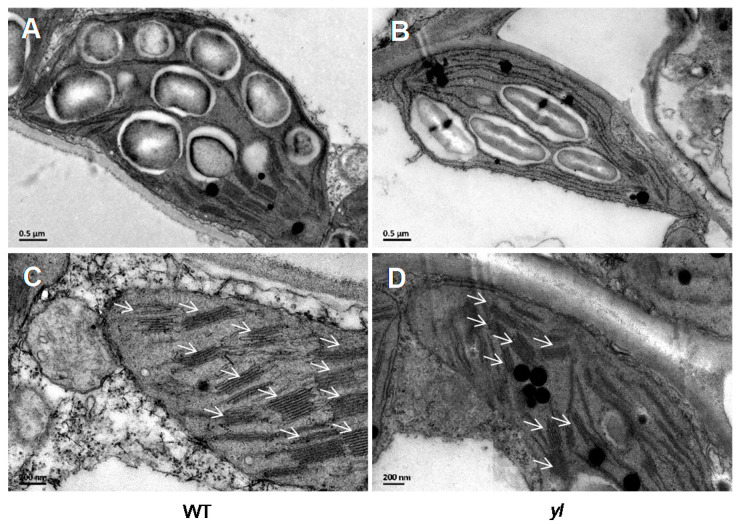
Chloroplast ultrastructure in the wild type and *yl* leaves. (**A**,**B**) Transmission electron micrographs (TEM) of chloroplasts in the wild type (**A**) and *yl* (**B**) leaves. (**C**,**D**) Magnified images of a chloroplast in the wild type (**C**) and *yl* (**D**) leaves. Arrows show grana. Bar = 0.5 μm in (**A**,**B**) and 200 nm in (**C**,**D**).

We further performed transmission electron microscopy (TEM) to compare the chloroplast ultrastructure between wild type and *yl* leaves. No differences in the number of chloroplasts per cell or chloroplast size were detected between the wild type and *yl*. However, the number of grana (stacked thylakoids) were slightly reduced in the *yl* chloroplasts (Figure 2A–D), indicating that *yl* chloroplasts were probably less functional than wild type chloroplasts.

### 2.2. Fine Mapping of the YL Gene

To unravel the molecular mechanism of the *yl* phenotype, we performed genetic mapping to isolate the *YL* gene. The *yl* mutant was crossed to two soybean cultivars with normal green leaves, Williams 82 and Zhonghuang 13, and three F_2_ mapping populations were produced by reciprocal crosses, *yl* × Williams 82, *yl* × Zhonghuang 13 and Zhonghuang 13 × *yl*. The leaf color of all F_1_ plants was normal green. The segregation ratio of green to yellow leaves appeared to be 3:1 in F_2_ populations, indicating that the *yl* phenotype is controlled by a single recessive nuclear gene (Table 1).

**Figure 3:**
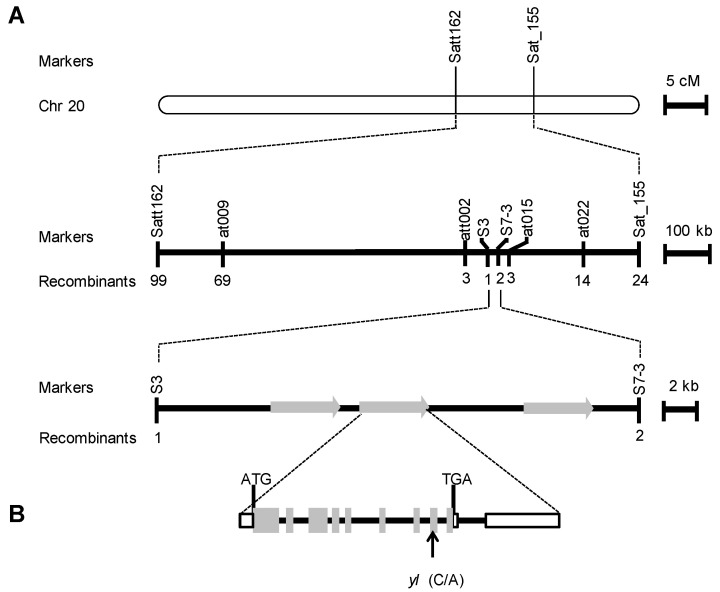
Fine mapping of the *YL* gene. (**A**) The *YL* locus was initially mapped to a region between markers Satt162 and Sat_155 on soybean chromosome 20. The gene was finally delimited to a 28 kb region between markers S3 and S7-3. Three predicted open reading frames (ORFs) were within this region. (**B**) *YL* (*Glyma.20G187000*) structure indicating nine exons (gray boxes), eight introns (line segments between the exons), and 5′ and 3′ untranslated regions (white boxes with black frame). Start (ATG) and stop (TGA) codons are marked. The *yl* mutation in the *YL* gene is shown.

The *yl* mutation was primarily mapped on chromosome 20 between microsatellite markers Satt162 and Sat_155, which were 9.9 centimorgans (cM) and 1.1 cM from *yl*, respectively (Figure 3A). The *YL* locus was ultimately fine mapped into a 28-kb interval between single nucleotide polymorphism (SNP) markers S3 and S7-3 (Figure 3A). According to the soybean gene annotation database (www.phytozome.net) [31], there were three putative open reading frames (ORFs) within this 28-kb region (Figure 3A). We sequenced the 28-kb sequences between the wild type and *yl* mutant and found a C to A transition at the eighth exon of *Glyma.20G187000* (Figure 3B).

### 2.3. YL Encodes an Organelle RNA Recognition Motif-Containing Protein 1 (GmORRM1)

**Figure 4:**
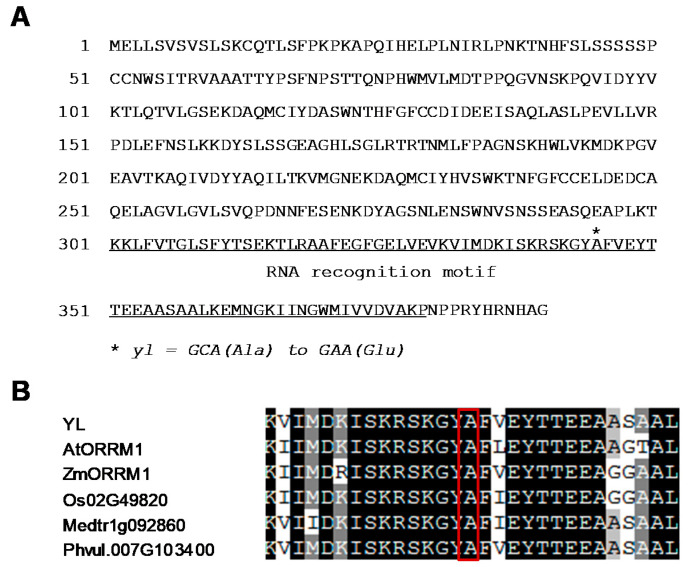
The YL protein contains an RNA recognition motif. (**A**) Amino acid sequence of YL. Position of the *yl* (asterisk) mutation is exhibited. The RNA recognition motif is underlined. (**B**) Partial alignment of the amino acid sequences of YL and its homologous proteins. The aligned sequences are from *Glycine max* (YL, Glyma.20G187000), *Arabidopsis thaliana* (AtORRM1, At3G20930), *Zea mays* (ZmORRM1, GRMZM5G899787), *Oryza sativa* (Os02g49820), *Medicago truncatula* (Medtr1g092860) and *Phaseolus vulgaris* (Phvul.007G103400). The red rectangle represents the conserved alanine residue in the RNA recognition motif.

The *YL* gene (*Glyma.20G187000*) encodes a protein of 390 amino acids with a C-terminal RNA recognition motif (Figure 4A). The *yl* mutation occurred in the RNA recognition motif and resulted in the substitution of an alanine with a glutamic acid (Figure 4A). The alignment of amino acid sequences homologous to YL from several plant species showed that this alanine residue in the RNA recognition motif was highly conserved (Figure 4B). In addition, the YL sequence shared 62.3% similarity with *Arabidopsis* ORRM1 (AtORRM1, At3G20930) and 58.2% similarity with maize ORRM1 (ZmORRM1, GRMZM5G899787), suggesting that YL (GmORRM1) and its homologs might have conserved functions.

### 2.4. Expression Pattern and Protein Subcellular Localization of YL

**Figure 5:**
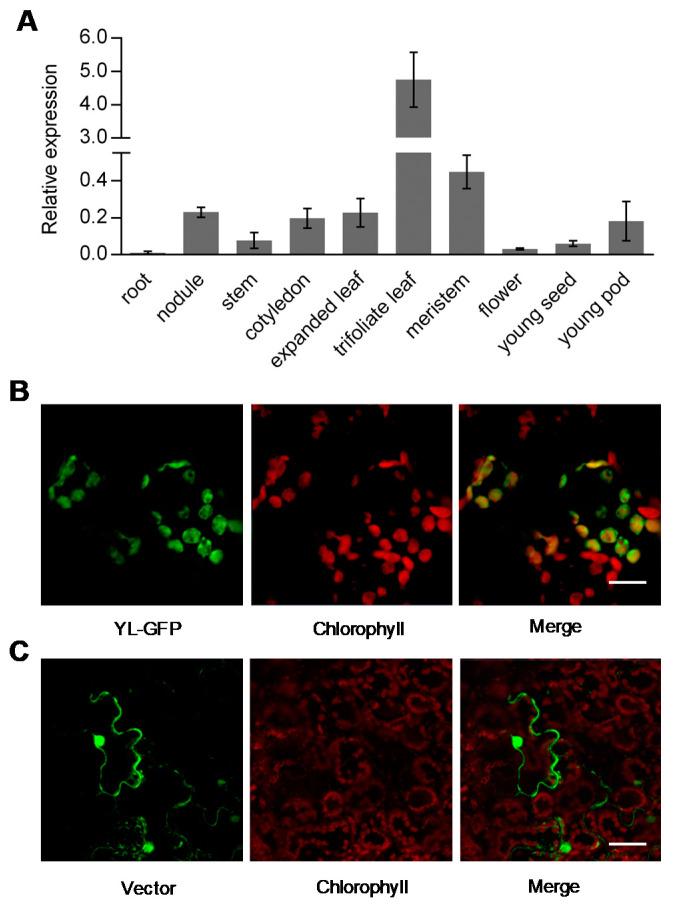
Expression pattern and subcellular localization of *YL*. (**A**) Relative expression levels of the *YL* gene in various soybean tissues. The real-time RT-PCR assays were performed in three biological replicates. qRT-PCR values for *YL* are normalized with the actin and ATP synthase genes. Error bars represent the SD. (**B**,**C**) Subcellular localization of the YL-GFP fusion protein (**B**) and GFP (**C**) in epidermal cells of *Nicotiana benthamiana* leaves. Bar = 20 µm in (**B**) and 50 µm in (**C**).

We performed quantitative real-time RT-PCR (qRT-PCR) to examine the expression of *YL* among various tissues. *YL* was expressed in all tissues tested, with the highest expression in trifoliate leaves, intermediate expression in meristem, nodules, expanded leaves, cotyledons, and young pods and weak expression in roots, stems, flowers, and young seeds (Figure 5A). The expression pattern of *YL* was consistent with the function of *YL* in affecting leaf color.

Using the Predotar and TargetP programs, the YL protein was predicted to localize in chloroplasts [32,33]. To obtain experimental verification of this localization, we expressed a YL-GFP fusion protein under the 35S promoter in *Nicotiana benthamiana* leaves. The green fluorescence signals of the YL-GFP fusion protein were observed in the chloroplasts of epidermal cells (Figure 5B,C).

### 2.5. Dramatic Defects of Chloroplast RNA Editing in yl

To further understand the function of *YL*, we performed DNA resequencing and RNA sequencing (RNA-seq) for the wild type to screen out soybean chloroplast editing sites, obtaining 44 predicted sites from 22 chloroplast transcripts (Table 2, Tables S1 and S2). In addition, the *rpl23*-89 site was identified through comparative analyses with *Arabidopsis* chloroplast editing sites (Table 2). Most of these sites were verified by direct sequencing of PCR products of transcripts or the corresponding genomic DNA carrying them (Table S2). Among the 45 editing sites, 44 sites were C-to-U conversions, 43 sites were in the coding regions of genes, and most of them caused alteration of the encoded amino acids (Table 2).

**Figure 6:**
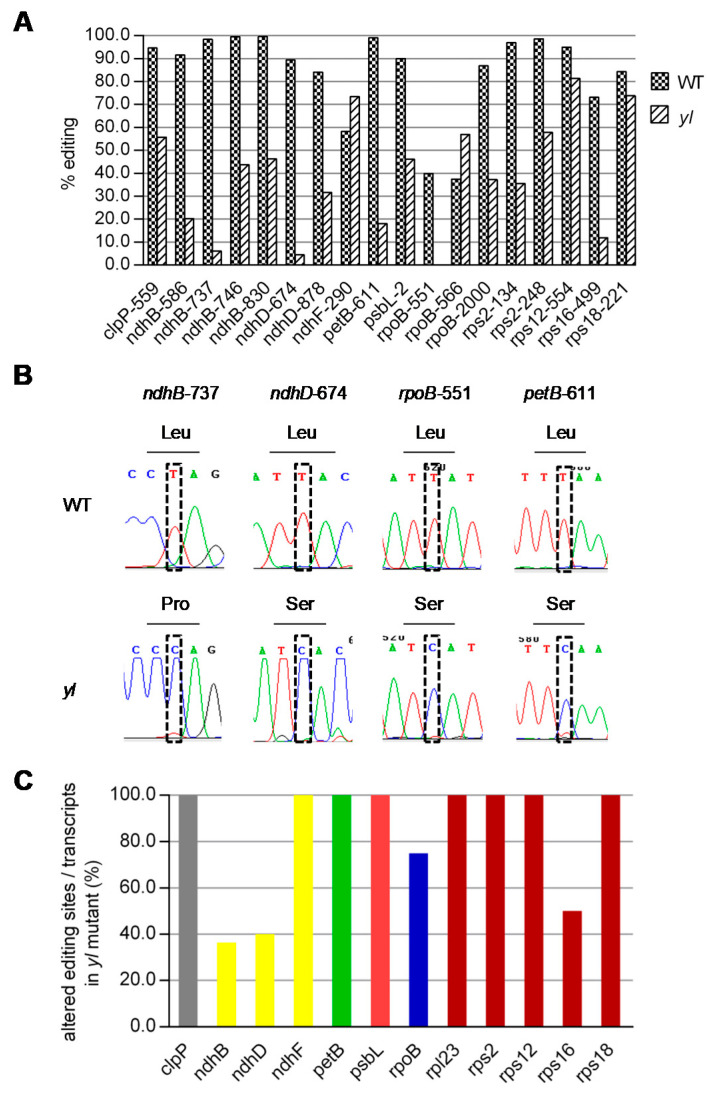
RNA editing at multiple chloroplast sites is impaired in the *yl* mutant. (**A**) Eighteen sites exhibit a significant alteration in editing of more than 10% in the *yl* mutant through RNA-seq analysis. (**B**) The three abolished editing sites and *petB*-611 in the *yl* mutant. Rectangular frames indicate defective editing sites. The corresponding amino acids are underlined. (**C**) Percentage of altered editing sites/transcripts in the *yl* mutant. Each bar shows a transcript color-coded in accordance with the complex to which it belongs.

The RNA-seq method was combined with direct sequencing of PCR products of transcripts carrying chloroplast RNA editing sites to compare the RNA editing between wild type and *yl* leaves. The results showed that the editing was completely abolished for *ndhB*-737, *ndhD*-674 and *rpoB*-551 in the *yl* mutant (Figure 6A,B, Tables S1 and S2). These deficiencies caused changes in the encoded amino acid residues from Leu, Leu and Leu in the wild type to Pro, Ser and Ser in the *yl* mutant, respectively. In addition, the editing of 14 sites was decreased by 10% to 90% in the *yl* mutant (Figure 6A, Tables S1 and S2). However, the *ndhF*-290 and *rpoB*-566 sites exhibited higher editing levels in the *yl* mutant compared with the wild type (Figure 6A, Tables S1 and S2).

We then investigated the distribution of the affected editing sites in the *yl* mutant. The 19 affected editing sites were distributed in 12 chloroplast transcripts encoding components of the Clp protease proteolytic subunit, NDH complex, cytochrome *b_6_f* complex, PSII complex, RNA polymerase or ribosomal proteins. As shown in Figure 6C, the percentage of altered editing sites per transcript varied from 36.4% to 100%, suggesting that the effect of the *YL* mutation on editing was site specific but not transcript specific.

## 3. Discussion

In plants, characterization of yellow foliar mutants will present an interesting opportunity to understand the complex photosynthesis process. The *yl* mutant reported here showed reduced Chl *a*, *b* and total Chl contents (Figure 1B). Our study identified a point mutation in the *YL* gene responsible for the mutant phenotype (Figure 3B). In addition, we discovered decreased PSII activity and a decreased net photosynthesis rate of *yl* leaves (Figure 1D,E). These results reveal that *YL* plays an important role in the photosynthetic process. This conclusion is supported by two pieces of evidence: (1) Strong expression of *YL* was detected in the trifoliate leaves; (2) The *yl* mutant lacked stacked thylakoids in the chloroplast, where photosynthesis takes place. The YL protein was homologous to maize ORRM1. In addition, the *Mu* transposon insertional mutant in *ZmORRM1* showed defects in the major photosynthetic enzyme complexes [20]. However, it is not known whether YL could influence similar photosynthetic proteins.

Based on bioinformatic analysis and protein subcellular localization experiments, we conclude that *YL* encodes a 390-amino-acid chloroplast-localized protein (Figure 4A and Figure 5B,C). Identification of the C-terminal RNA recognition motif of YL and the high level of homology with AtORRM1 and ZmORRM1 strongly suggest that YL functions in RNA editing. ORRM1 controlled the extent of editing in 62% of the chloroplast sites in *Arabidopsis* and 81% of sites in maize [20]. In our study, YL was required for the editing of 42% of chloroplast sites in 12 chloroplast transcripts (Figure 6A, Tables S1 and S2). Moreover, we compared RNA editing alterations in soybean, *Arabidopsis* and maize *orrm1* mutants. Notably, the three completely lost editing sites, *ndhB*-737, *ndhD*-674 and *rpoB*-551, in the *yl* mutant also exhibited a pronounced reduction in editing in *Arabidopsis* or maize *orrm1* mutants (Figure 6A,B, Tables S1 and S2). In contrast, the editing of *ndhF*-290 exhibited no change in the *Arabidopsis orrm1* mutant, whereas we observed a slight increase in the editing of *ndhF*-290 in the *yl* mutant (Figure 6A, Table S1). Consequently, it seems that ORRM1 showed species-specific functions in plants. These results suggest that YL may be involved in chloroplast RNA editing.

Twelve chloroplast transcripts, which encode components of the Clp protease proteolytic subunit, NDH complex, cytochrome *b_6_f* complex, PSII complex, RNA polymerase or ribosomal proteins, could not be edited in the *yl* mutant (Figure 6C). The RNA editing defect in transcripts encoding the components of the Clp protease proteolytic subunit, NDH complex, and RNA polymerase or ribosomal proteins may not be the main reason for the *yl* mutant phenotype. Although the *Arabidopsis orrm1* mutant exhibited severe editing defects in transcripts encoding these proteins, it did not show any phenotypic deficiencies [20]. In addition, partial knockout of *clpP* in tobacco resulted in an asymmetric, slender leaf shape but normal leaf color [34]. Moreover, no obvious phenotype was observed in tobacco mutants with disrupted NDH complexes [35].

The reduction in *petB*-611 and *psbL*-2 editing may have large contributions to the *yl* phenotype. The *petB* gene encodes cytochrome *b_6_*, which is one of the major subunits of the cytochrome *b_6_f* complex mediating electron transfer between PSII and I [36,37,38]. In the *yl* mutant, defective editing at *petB*-611 resulted in a change from the wild type residue at position 204 (Leu) to Ser (Figure 6B). The Leu^204^ residue belongs to the D helix span, which is involved in heme binding [37,39]. Leu^204^ to Ser^204^ alteration likely disrupts the assembly of cytochrome *b_6_f* complexes, as suggested by the behavior of *petB* mutants in *Chlamydomonas reinhardtii* [37]. The *yl* mutant is phenotypically similar to a tobacco *petB* mutant in which the portion of the *petB* coding region was replaced with an *aadA* cassette [38]. Both *yl* and *petB* mutants display yellow (pale green) leaf color and reduced chlorophyll contents and grana stacks. The *psbL* gene encodes a conserved low molecular weight protein of PS II [40]. In wild type soybean, the initiator codon (AUG) of *psbL* is formed by a C to U editing of the ACG codon. In the present *yl* mutant, defective editing at *psbL*-2 may partially influence the formation of the initiator codon (AUG). The lack of *PsbL* in tobacco has been implicated as impairing the assembly of PSII [41,42]. The reduced Fv/Fm in the *yl* mutant is similar to the behavior of *Arabidopsis* mutants deficient in RNA editing sites in plastid transcripts encoding PSII proteins, including *orrm6* mutants [21].

The cytochrome *b_6_f* and PSII complexes belong to the main components of the photosynthetic electron transfer chain [2]. The *yl* mutant, exhibiting RNA editing deficiency at *petB*-611 and *psbL*-2, may be unable to assemble functional cytochrome *b_6_f* and PSII complexes. Since the localization of cytochrome *b_6_f* and PSII complexes is predominantly in the grana regions of the thylakoid membrane system, this inference may account for lacked grana in *yl* chloroplast. In soybean, some mutants with defects in photosynthetic electron carrier proteins display a yellow phenotype [4,8]. For instance, the mutation in the PSII extrinsic protein GmpsbP leads to a lethal-yellow phenotype, extremely low Fv/Fm, and failure of proplastid differentiation into normal chloroplasts with grana [8]. The *yl* mutant showed reduced Fv/Fm, indicating that the *yl* mutant cannot utilize the absorbed light in photochemistry as effectively as the wild type. When more light energy is absorbed than is converted in plants, the photosynthetic organism is subjected to photooxidative stress, known as photoinhibition, leading to pigment bleaching, inactivation of electron transport and damage of the reaction center [43,44]. A recent study has shown that a leaf yellowing mutant phenotype in soybean may be largely due to the abnormal light absorption in the photosynthesis process [45]. Thus, photoinhibition may also be the cause of decreased Chl contents and net photosynthesis rate in the *yl* mutant.

## 4. Materials and Methods

### 4.1. Plant Materials and Growth Conditions

Seeds of soybean cultivar Jindou 23 (wildtype) were mutagenized with ethyl methane sulfonate (EMS). The phenotypes of the second mutant generation (M_2_ progeny) plants were observed and the *yl* mutant with yellow foliage was identified from the M_2_ progeny. The *yl* mutant has been continuously self-pollinated and selected based on the yellow foliar phenotype. For phenotype characterization of the *yl* mutant, the simultaneous field trial consisted of three replicates per genotype, planted in 0.50 m row spacing and 0.10 m spacing in the rows. To analyze the inheritance pattern of the *yl* mutant and fine-mapping of the *YL* gene, three F_2_ populations were generated from reciprocal crosses between the *yl* mutant and two soybean cultivars, Williams 82 and Zhonghuang 13 (*yl* × Williams 82, *yl* × Zhonghuang 13 and Zhonghuang 13 × *yl*). All soybean materials were planted in the experimental field at the Institute of Genetics and Developmental Biology, Chinese Academy of Sciences (Beijing, China), during the natural growing seasons (May to October). The common meteorological conditions during soybean growing seasons in Beijing were as follows: (1) mean daily temperatures: about 24 °C. (2) overall precipitation: 400 ~ 600 mm.

### 4.2. Chl Contents, Net Photosynthetic Rate and Photochemical Efficiency Analysis

The third true leaves from the top of five-week-old soybean plants were used to estimate Chl contents, net photosynthetic rate and photochemical efficiency. The total Chl, Chl *a* and Chl *b* contents were determined as previously described [46]. Fresh leaves (200 mg) were immersed into 10 mL of 95% ethanol for pigments extraction under dark conditions. Then the absorbance of supernatant was quantified spectrophotometrically at 665 and 649 nm. Chlorophyll contents were calculated using the following formulas [46]: Chl *a* (mg/g) = [(13.95OD_665_ − 6.88OD_649_) × 10]/(tissue fresh weight (g) × 1000); Chl *b* (mg/g) = [(24.96OD_649_ − 7.32OD_665_) × 10]/(tissue fresh weight (g) × 1000); Total Chl (mg/g) = Chl *a* (mg/g) + Chl *b* (mg/g). Net photosynthetic rate was measured using an Li-6400 instrument (LI-COR, Lincoln, NE, USA) under a CO_2_ concentration of 250 μmol mol^−1^ and 1000 μmol m^−2^s^−1^ photosynthetically active radiation (PAR) between 9:00 and 11:00 in the morning. The maximum photochemical efficiency of photosystem II was measured as the Fv/Fm using an IMAGING-PAM Chlorophyll Fluorometer (Heinz Walz, Effeltrich, Germany).

Three to eight individual plants were examined for each material and the examination were repeated at least three times. The experimental data for each material are reported as mean ± standard deviation (SD). Statistical significance of differences between *yl* mutant and wildtype plants were tested using independent sample Student’s t test algorithm. All statistical analysis were performed in SPSS Statistics 17.0.

### 4.3. Transmission Electron Microscopy

The third leaves of 30-day-old wild type and *yl* plants were collected and fixed for at least 2 h at 4 °C in 0.1 M phosphate buffer, pH 7.4, with 2.5% glutaraldehyde and washed with the same buffer. The samples were fixed in 1% osmium tetroxide for 2 h and then dehydrated through an ethanol gradient, infiltrated and embedded in epoxy resin. Ultra-thin sections were obtained using a diamond knife and mounted on copper grids. Then the grids were stained with uranyl acetate and lead citrate, and examined using a JEM-1400 (JEOL, Tokyo, Japan) transmission electron microscope.

### 4.4. Fine Mapping of the YL Gene

Three F_2_ populations generated from reciprocal crosses between the *yl* mutant and two soybean cultivars, Williams 82 and Zhonghuang 13 (*yl* × Williams 82, *yl* × Zhonghuang 13 and Zhonghuang 13 × *yl*), were used for genetic analysis. In the F_2_ populations, genomic DNA was isolated from selected etiolated seedlings exhibiting mutant phenotype for gene mapping. The cetyl trimethyl ammonium bromide (CTAB) method was used for genomic DNA extraction from the fresh young trifoliate leaf tissue and for all subsequent DNA extractions using this method unless otherwise stated. Briefly, frozen tissue was powdered and dispersed in 2 × CTAB extraction buffer, and incubated at 65 °C for about 60 min. Chloroform/Tris-phenol, 1:1 (vol/vol), was added and mixed to form an emulsion that was centrifuged for 10 min. The upper aqueous phase was transferred to a new tube, and 2/3 vol of isopropanol was added for DNA precipitation. The extracted DNA was treated with RNase to remove RNA contamination.

For primary mapping, 92 mutant individuals were selected from two F_2_ populations between *yl* and Williams 82 or Zhonghuang 13. The markers used for primary mapping were 71 published SSR markers (http://soybase.org/) [47]. A total of 770 mutant individuals selected from the F_2_ population between the *yl* mutant and Williams 82 were used for fine mapping. New molecular markers were developed for fine mapping (Table S3). To identify the candidate gene, the corresponding DNA fragments within the fine mapping region were amplified from the wild type and *yl* mutant using special primers (Table S3) and sequenced.

### 4.5. RNA Extraction and Quantitative Real-Time RT-PCR

For analysis of the expression of *YL* in various soybean tissues, total RNA was isolated from root, nodule, stem, cotyledon, expanded leaf, trifoliate leaf, meristem, flower, young seed and pod collected from three individuals. For the analysis of chloroplast RNA editing in the *yl* mutant and wild type, total RNA was isolated from trifoliate leaves of five-week-old soybean plants. Fresh tissue was frozen immediately in liquid nitrogen and ground to powder using a mortar and pestle. Total RNA was extracted using 1 mL TRIzol reagent (Invitrogen Life Technologies, Carlsbad, CA, USA), according to the manufacturer’s instructions. After ethanol precipitation, the RNA was dissolved in RNase-free water.

Approximately 2 μg of total RNA were reverse transcribed using the Prime Script RT reagent Kit with gDNA Eraser (TaKaRa, Beijing, China), according to the manufacturer’s instructions. Quantitative real-time RT-PCR was performed in a 20 μL reaction mix containing 50 ng of cDNA, 1 × Light Cycler 480 SYBR Green I Master (Roche, Mannheim, Germany), 0.5 μM of each primer on a Lightcycler 480 (Roche, Mannheim, Germany) machine using the following PCR profile: 2 min at 94 °C, followed by 40 cycles of 15 s at 94 °C, 15 s at 60 °C, and 30 s at 72 °C. The dissociation curve analysis was conducted to verify the PCR specificity. Three biological replicates with three technical replicates were analyzed to quantify the levels of gene expression. The soybean actin and ATP synthase genes were used as internal standards to normalize the expression of *YL* using the 2^‒ΔCt^ method. The primers used for quantitative real-time RT-PCR were designed using the Primer Premier 5 software (Premier, San Francisco, CA, USA) and listed in Table S3.

### 4.6. Subcellular Localization

To determine its subcellular localization, the coding sequence of *YL* lacking its stop codon was amplified by PCR in a 50 μL volume that contained 200 ng cDNA from wild type, 1 × PCR buffer, 0.4 mM dNTPs, 1 U KOD FX Neo (Toyobo, Osaka, Japan) and 0.3 μM of both forward and reverse primers of *YL-GFP* listed in Table S3. The sample was heated to 94 °C for 2 min, followed by 45 cycles of denaturation at 94 °C for 15 s, annealing at 61 °C for 30 s, elongation at 68 °C for 1 min. The amplified product was cloned into the pCAMBIA 1302 vector between the cauliflower mosaic virus 35S promoter and the GFP-coding sequence. The construct of YL-GFP vector was sequencing confirmed by Sangon Biotech (Shanghai, China).

For transformation of *Agrobacterium*, 1 μg purified plasmid DNA was added to competent cells of thawing *Agrobacterium tumefaciens* strain *GV3101* on ice. After ice bath for 30 min, the cell/DNA mix was immersed in liquid nitrogen for 1 min and subsequently incubated at 37 °C for 5 min, then ice bath for 2 min. By adding 900 μL liquid growth medium (no antibiotics), the cell/DNA mix was shocked for at least 120 min at 28 °C. After centrifugation, the cells were resuspended and plated on an agar plate containing kanamycin (50 mg/L) for selection of transformants.

For infiltration of *Nicotiana benthamiana*, the *Agrobacterium tumefaciens* strains carrying the YL-GFP, pCAMBIA 1302 and p19 of tomato bushy stunt virus plasmids were grown at 28 °C in liquid growth medium with kanamycin (50 mg/L), rifampicin (50 mg/L) and gentamicin (25 mg/L) until OD_600_ reached 1.0. The *Agrobacterium tumefaciens* strains containing the YL-GFP and p19 or pCAMBIA 1302 and p19 plasmids were mixed. After centrifugation of the strain mixtures, the harvested cells were resuspended in 10 mM MES buffer containing 10 mM MgCl_2_ and 100 mM acetosyringone to a final OD_600_ of 1.0, followed by incubation at room temperature for 120 min. Strain mixtures were infiltrated into the abaxial surface of leaves of four-week-old *Nicotiana benthamiana* plants using a 1 mL syringe. The transformed epidermal cells were detected using a Zeiss LSM710 confocal microscope (Carl Zeiss Microscopy GmbH, Jena, Germany).

### 4.7. Chloroplast RNA Editing Analysis

For chloroplast RNA editing analysis, total DNA and RNA were isolated from wild type and *yl* leaves as described in Section 4.4 and Section 4.5. Next, the DNA and RNA were submitted to Berry Genomics (Beijing) for DNA resequencing and rRNA-depleted strand-specific RNA-seq, respectively. For RNA-seq analysis, four cDNA libraries (two biological replicates per genotype) were constructed. All sequencing data used in this research were deposited in the National Center for Biotechnology Information (NCBI) Sequence Read Archive under BioProject ID PRJNA616185.

Clean reads were quality checked using FastQC (version 0.11.3) (http://www.bioinformatics.babraham.ac.uk/projects/fastqc/) [48]. After quality control, reads were aligned to the soybean chloroplast genome (ncbi.nlm.nih.gov/nuccore/DQ312375.1) [49] using HISAT (version 2.0.0) [50]. SNP calling was performed by GATK combined with Samtools [51,52]. SNPs with read numbers ≥ 20 from the RNA-seq and the corresponding DNA resequencing data were compared and the different chloroplast allele bases between the two data sets were considered as the candidate RNA editing sites. Because C-to-U editing in plastid and mitochondrial mRNAs appear to be ubiquitous in land plants, the editing efficiency of each editing site was calculated using the following equation: Editing (%) = U/(C + U) × 100. U represents the read number of SNPs that are different from DNAs in plastid mRNAs. C represents the read number of SNPs that are identical as DNAs in the same plastid editing site.

To experimentally validate the RNA editing sites derived from RNA-seq, the genomic and transcript regions surrounding these sites were amplified from another wild type biological replicate and *yl* mutant biological replicate using KOD FX High-Fidelity DNA polymerase (Toyobo, Osaka, Japan). The PCR products were sequenced and compared to identify SNP changes resulting from RNA editing. The RNA editing extent was estimated by the relative heights of the peaks of the nucleotide in the sequence analyzed. The primer sequences are listed in Table S3.

## 5. Conclusions

In this study, we characterized one *yellow leaf* mutant and identified *YL*, a soybean homolog of *Arabidopsis ORRM1* that is involved in RNA editing. The *yl* mutant displayed yellow leaves, reduced chlorophyll contents, impaired photosynthesis and an altered chloroplast ultrastructure. Through fine mapping, the *yl* mutation was narrowed down to a 28-kb genomic region in chromosome 20 between markers S3 and S7-3. DNA sequencing revealed that the *YL* mutation in the *yl* mutant is a C to A transition in *Glyma.20G187000*, which causes an amino acid alteration. Further function analysis of *YL* uncovered that the point mutation in *yl* influenced the editing extent in 42% of the chloroplast sites surveyed. However, we speculate that only the decreased level of conversion of cytidine to uridine at *petB*-611 and *psbL*-2 may affect normal photosynthesis in *yl* leaves. Together, our findings indicate that soybean YL protein influences photosynthesis, possibly via its function in chloroplast RNA editing.

## Figures and Tables

**Table 1:** Segregation pattern and chi-square tests for green/yellow leaves of F_2_ progeny from the crosses between *yl* and Williams 82 or Zhonghuang 13.

Crosses	F_1_	Number of F_2_ Plants			χ^2^ (3:1)	*P*
		Wild Type	Mutant	Total		
*yl* × Williams 82	Normal	679	227	906	0	>0.9
*yl* × Zhonghuang13	Normal	440	163	603	1.221	0.50~0.25
Zhonghuang13 × *yl*	Normal	448	132	580	1.437	0.25~0.10

**Table 2:** RNA editing sites in soybean chloroplast transcripts.

Gene	Position ^a^	Codon Site ^b^	Conversion
*accD*	617	2	S(uCg)→L(uUg)
*atpF*	92	2	P(cCa)→L(cUa)
*clpP*	559	1	H(Cau)→Y(Uau)
*ndhA*	341	2	S(uCa)→L(uUa)
	1073	2	S(uCu)→F(uUu)
*ndhB*	9	3	W(ugG)→stop codon (ugA)
	149	2	S(uCa)→L(uUa)
	542	2	T(aCg)→M(aUg)
	586	1	H(Cau)→Y(Uau)
	737	2	P(cCa)→L(cUa)
	746	2	S(uCu)→F(uUu)
	830	2	S(uCa)→L(uUa)
	836	2	S(uCa)→L(uUa)
	1112	2	S(uCa)→L(uUa)
	1255	1	H(Cau)→Y(Uau)
	1481	2	P(cCa)→L(cUa)
*ndhC*	323	2	S(uCa)→L(uUa)
*ndhD*	2	2	T(aCg)→M(aUg)
	383	2	T(aCa)→I(aUa)
	674	2	S(uCa)→L(uUa)
	878	2	S(uCa)→L(uUa)
	1298	2	S(uCa)→L(uUa)
*ndhE*	233	2	P(cCg)→L(cUg)
*ndhF*	290	2	S(uCa)→L(uUa)
*petB*	611	2	S(uCa)→L(uUa)
*psaI*	79	1	H(Cau)→Y(Uau)
*psbE*	214	2	S(uCc)→F(uUc)
*psbF*	6	3	T(acC)→T(acU)
	77	2	S(uCu)→F(uUu)
*psbL*	2	2	T(aCg)→M(aUg)
*rpl23*	89	2	S(uCa)→L(uUa)
*rpoA*	200	2	S(uCa)→L(uUa)
*rpoB*	338	2	S(uCu)→F(uUu)
	551	2	S(uCa)→L(uUa)
	566	2	S(uCg)→L(uUg)
	2000	2	S(uCu)→F(uUu)
*rpoC1*	41	2	S(uCa)→L(uUa)
	488	2	S(uCa)→L(uUa)
*rps2*	134	2	T(aCa)→I(aUa)
	248	2	S(uCa)→L(uUa)
*rps12*	554 ^c^		
*rps14*	80	2	S(uCa)→L(uUa)
*rps16*	499 ^c^		
	212	2	S(uCa)→L(uUa)
*rps18*	221	2	S(uCg)→L(uUg)

^a^ Position is given with respect to the initiation codon of each chloroplast transcript. ^b^ Codon site is the order in the amino acid codon. ^c^ RNA editing sites are in introns of the chloroplast gene. The position here is given with respect to the initiation codon of each gene.

## Supplementary Materials

Supplementary materials can be found at https://www.mdpi.com/1422-0067/21/12/4275/s1. Table S1. Comparison of the extent of RNA editing of chloroplast sites between the wild type and *yl* mutant by RNA-seq. Table S2. Verification of the extent of chloroplast RNA editing by Sanger sequencing. Table S3. Primers used in this study.
