# Supplementary material for: Mutation of YL Results in a Yellow Leaf with Chloroplast RNA Editing Defect in Soybean
Source: Int J Mol Sci. 2020 Jun 16;21(12):4275. doi: 10.3390/ijms21124275 (PMC7348699; doi:10.3390/ijms21124275)
Supplement: Supplementary file 1 [file ijms-21-04275-s001.pdf]

Table S1. Comparison of the extent of RNA editing of chloroplast sites between the wild type and *yl* mutant by RNA-seq.

**Gene**	**Position** [a]	**Wild type**		***yl***	
		**Coverage [reads]**	**Editing (%)**	**Coverage [reads]**	**Editing (%)**
accD	617	696	91.24	258	84.50
atpF	92	3531	97.73	2260	96.59
clpP	559	2338	94.65	1990	55.73
ndhA	341	689	91.29	450	94.00
	1073	2277	90.65	1553	90.79
ndhB	9	28	100.00	20	100.00
	149	50	96.00	28	96.43
	542	290	100.00	192	100.00
	586	95	91.58	74	20.27
	737	126	98.41	147	6.12
	746	175	99.43	153	43.79
	830	266	99.62	134	46.27
	836	267	100.00	78	100.00
	1112	189	96.83	71	100.00
	1255	727	99.72	209	98.56
	1481	138	100.00	50	100.00
ndhC	323	1231	95.69	636	97.96
ndhD	2	1686	55.28	1488	55.85
	383	1896	91.59	1532	92.95
	674	2597	89.37	1836	4.52
	878	3442	84.02	2354	31.61
	1298	2026	87.76	1376	94.40
ndhE	233	2342	89.75	1136	84.86
ndhF	290	645	58.29	192	73.44
petB	611	1260	99.05	3557	18.11
psaI	79	2311	94.50	884	87.78
psbE	214	1658	99.70	1132	99.65
psbF	6	1654	85.31	1289	92.47
	77	2694	94.51	1523	93.11
psbL	2	1239	89.99	700	46.14
rpoA	200	1430	73.01	1091	72.23
rpoB	338	132	68.18	187	66.84
	551	173	39.88	120	0.00
	566	160	37.50	121	57.02
	2000	145	86.90	161	37.27
rpoC1	41	556	96.22	497	97.99
	488	1054	47.25	1152	51.48
rps2	134	1566	96.87	817	35.50
	248	1875	98.56	967	57.81
rps12	554 [b]	139	94.96	48	81.25
rps14	80	1159	86.11	2641	89.10
rps16	499 [b]	350	73.14	260	11.92
	212	2662	95.79	643	91.91
rps18	221	1349	84.36	516	73.84

The coverage of each editing site is presented in reads. Editing (%) = U/(C + U) × 100.

[a] Position is given with respect to the initiation codon of each chloroplast transcript.

[b] The RNA editing site is in the intron of the plastid gene. The position here is given with respect to the initiation codon of the DNA sequence of each gene.

Table S2. Verification of the extent of chloroplast RNA editing by Sanger sequencing.

Name	WT		*yl*	
	Genomic DNA	cDNA	Genomic DNA	cDNA
atpF-92	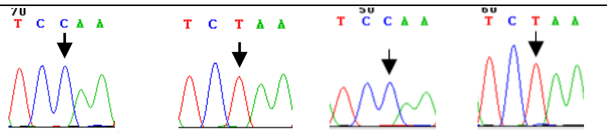			
clpP-559	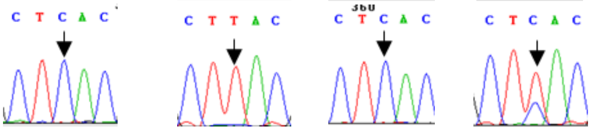			
ndhA-341	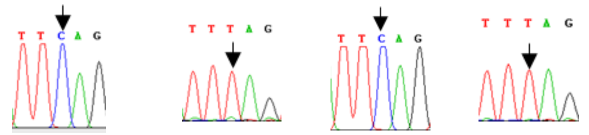			
ndhB-149	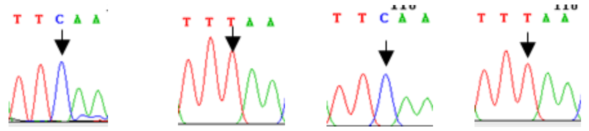			
ndhB-542	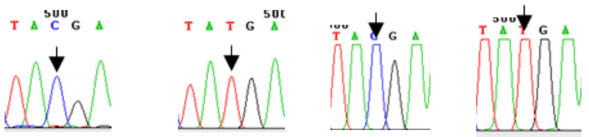			
ndhB-586	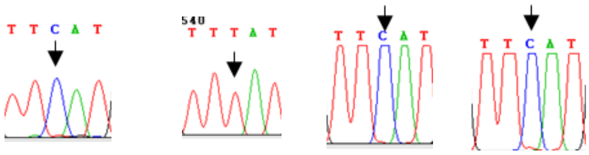			
ndhB-737	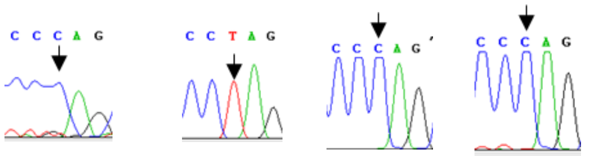			
ndhB-746	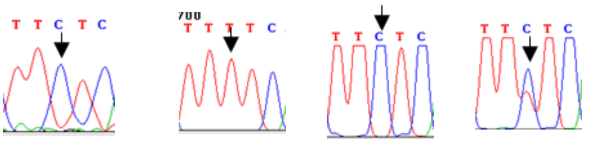			
ndhB-830	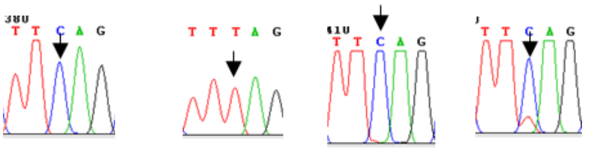			
ndhB-836	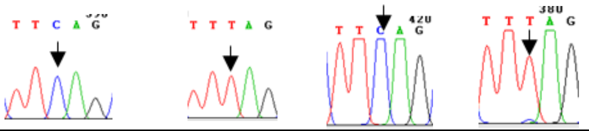			

Table S2. (continued).

Name	WT		*yl*	
	Genomic DNA	cDNA	Genomic DNA	cDNA
ndhB-1112	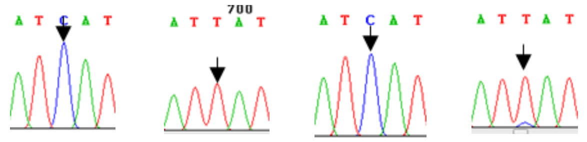			
ndhB-1255	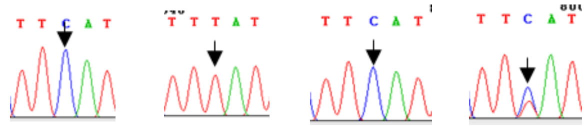			
ndhB-1481	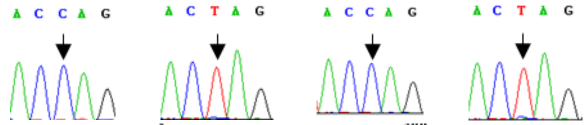			
ndhC-323	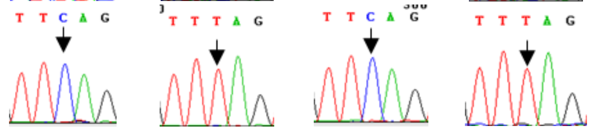			
ndhD-383	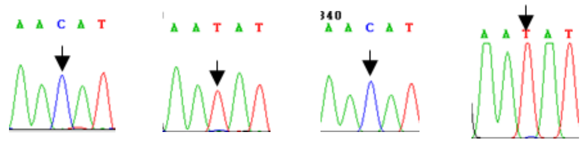			
ndhD-674	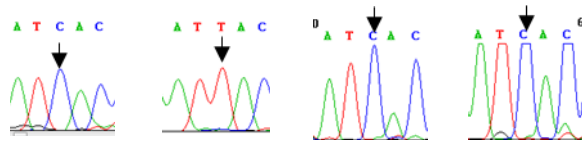			
ndhD-878	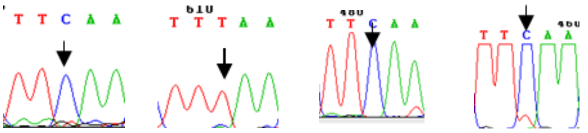			
ndhD-1298	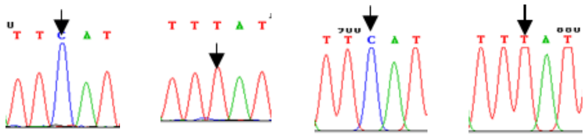			
petB-611	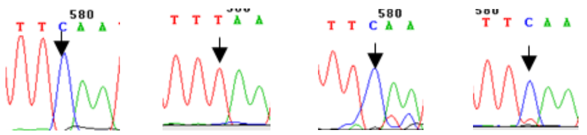			
psaI-79	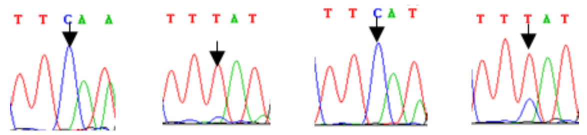			
psbE-214	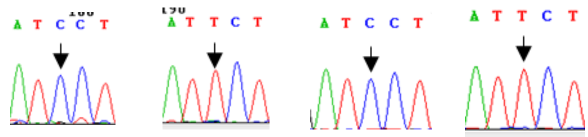			

Table S2. (continued).

Name	WT		*yl*	
	Genomic DNA	cDNA	Genomic DNA	cDNA
rpl23-89	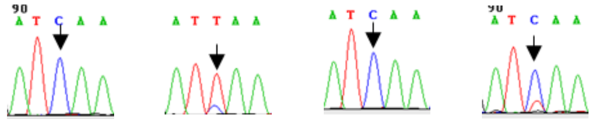			
rpoA-200	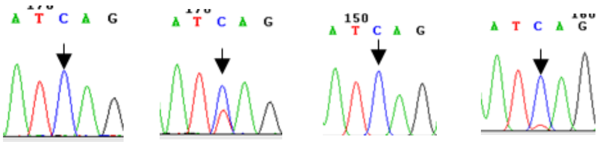			
rpoB-338	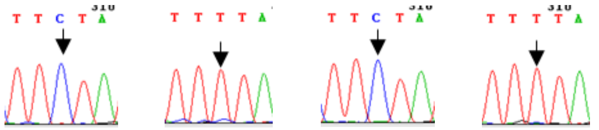			
rpoB-551	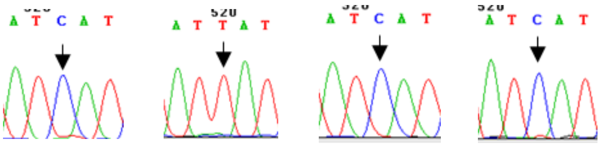			
rpoB-566	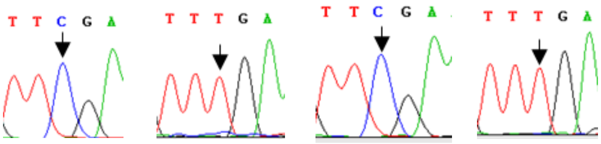			
rpoB-2000	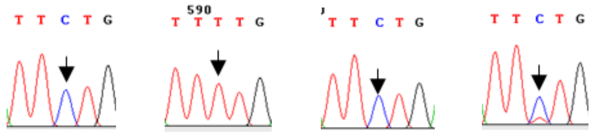			
rpoC1-41	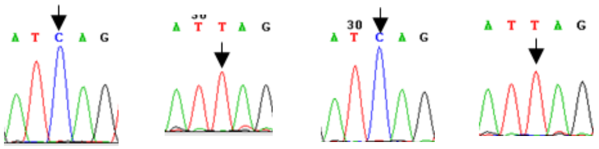			
rpoC1-488	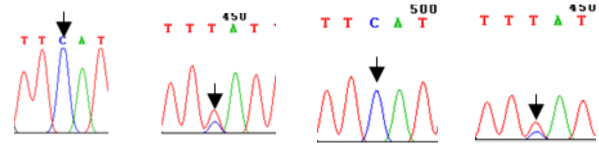			
rps2-134	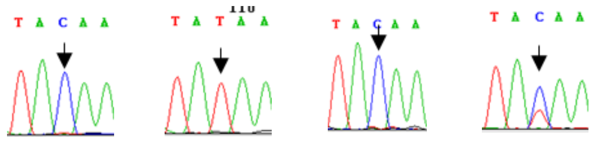			
rps2-248	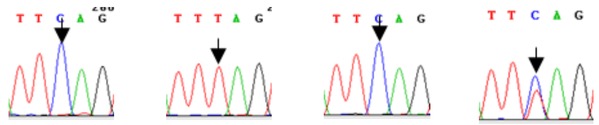			

Table S2. (continued).

Name	WT		*yl*	
	**Genomic DNA**	**cDNA**	**Genomic DNA**	**cDNA**
rps14-80	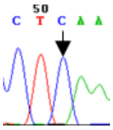	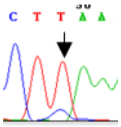	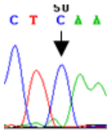	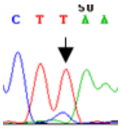
rps16-212	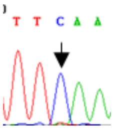	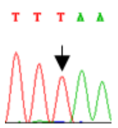	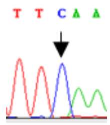	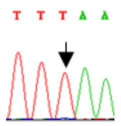
rps18-221	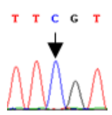	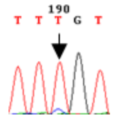	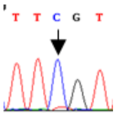	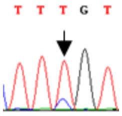

The sequence results were derived from PCR or RT-PCR products amplified from DNA or cDNA, separately. The editing sites are indicated by arrows.

Table S3. Primers used in this study.

**Primers used for fine mapping of *YL***		
**Name**	**Forward primers (5'-3')**	**Reverse primers (5'-3')**
at009	AATCCATTCACTCTCCCAAA	TCATCGCCAACATTTCACTA
att002	TTGCCCAAGGACTTCCACAT	GAACAAGCCCTTCGGAGATT
at015	AATAATGCACACGACTTAGGGT	TAATCAGCATCAGCACATACAA
at022	CGGAGCAAGTTGGTTTTGACTG	ATTATTTGGGGAATTGGGGTGT
S3	GTGATGCTGTTTCTTGCGAGTT	ATGAGTTATCCTGCTGCCTTGA
S7-3	AATACGATGGCGATAAGAAC	ACAGGAACAGGAGGAAGTGA

Primers used for determining the *YL* gene		
Name	Forward primers (5'-3')	Reverse primers (5'-3')
Seq28k-1	CGCAACTTGGTTCATCACTGTA	TGAAGGGAATAGGGAGAATCTC
Seq28k-2	GAGATTCTCCCTATTCCCTT	TACTCACATTTGCACCTTCG
Seq28k-3	AATACGATGGCGATAAGAAC	ACAGGAACAGGAGGAAGTGA
Seq28k-4	AAATACGTAATCGCATTCGAGT	GCAAAGCAGGAAATCAGATACA
Seq28k-5	CATGGCTACTTTCACCTTTA	TTCACCTTTCTCTCCTTCTA
Seq28k-6	ACTTTGTAGTTCTGTGCCTCTTG	AACATGCTGAGCTGGAATCTTAA
Seq28k-7	ATGACTTTAAGATTCCAGCTCAG	GGACATTTCCCTTCACTACCACC
Seq28k-8	CTCATAGATAACACTGGATAGGGAAG	TTTTAGGCACAAGGGCAAATAGAAC
Seq28k-9	CATGGCTACTTTCACCTTTA	ATTAAGATGCCATGCTGACA
Seq28k-10	CTTATCAAGTCACATGGACATTGTG	CTACAAGGCTAACCAGCAAGCTCTT
Seq28k-11	AGGTCATGGGAAAGTGAGGACAGTG	TGATTCCTGTGGTATCTTGGTGGGT
Seq28k-12	TGTTGAATACACCACAGAGGAGGCT	CAATAGGAACAACTCGAAGGGACTG
Seq28k-13	AATATTGTAGCCTCTATAGGAATCTGGC	GTCTTTTGTTGTTAGATCAACCCAAATA
Seq28k-14	TGAGATGGTCCCTGATGGAAAATAT	TCTGAAAGCTGACCCAGTAGTGGAA
Seq28k-15	ATTACTGGTCCTTTCTGCTTCAC	TAATAGCGTAAGTCTGCACAACAAG
Seq28k-16	GCCTCTTTTGTTATCATCTATGTCCTTG	TAGTTCAGACCCTACTTACTTTACTCTG

Seq28k-17	TCCCACTATTTAATGTGATATTTGC	AAACAACAAGACAAGAAGACTACCC
Seq28k-18	TGCCTACTCGTGGAATGTTG	TGAAGGTTTCGGGTTTGTTT
Seq28k-19	TTTTCTTATAGCGACTTGCC	CATCAGGGACCATCTCATTC
Seq28k-20	ATGTCATACTCCGACTTGTT	CATAGTTTCTGATATTTCCA
Seq28k-21	ACATTTCGCTCTATTCTGTTC	GACGGTTACTTGACTTCTTTCT
Seq28k-22	TTCATATTTGCGTTAATGATTTCTC	TCATTTCAAAGTGTTCATTGGATAC
Seq28k-23	ACTGCAATGAGTAATGCTGAGCCACC	GTCGTCGTCAAACCAAACCAAATCCTAT
Seq28k-24	GGCGGCATAGCATCGTCATCAT	TGGCGAAGGAGACTCTAGGAACT
Seq28k-25	GCTAAATGAGAAGCTAAACTTACCCTAA	CAAGACAAGAAGACTACCCTAAACTA
Seq28k-26	TCAAGTCTCAAGATACGCTTATTATCAT	CGTCAAACTTTAATTTTATTTCTTTCTG
Seq28k-27	AAAAGTATAGGGAAAGGGTGGGAGA	TTGGCATTTGCTAAGTGAGACAGAC
Seq28k-28	TTGAATGCTGTTGTCGTAATTTTCT	CCATAGGCAAAGCTTATACCGTATT
Seq28k-29	AACCTCCACGATACTTCCAGAACCT	TCAATCATCCAGAACAGGGAATCAG
Seq28k-30	AAATTGGTAGGAAGGTTACTTATGT	CAATGATTATCTTTTCTAGGATTTG
Seq28k-31	GTGGATCTTGGAGATAGAAATA	TTTCATTACTTGTACCGATTAC
Seq28k-32	GGGTCAAGGATGTGACAACGAT	CTGATGCCACTCAACCATGACA
Seq28k-33	ATTCACCAGGGCTTAACAAAGT	AAAAGAACGAAGGGAGTATCAT
Seq28k-34	CACCGCCTCTAATACTACGCTA	GGCGAAGGAGACTCTAGGAACT
Primers used for vector construction		
Name	Forward primers (5’-3’)	Reverse primers (5’-3’)
YL-GFP	TGCCATGGCAATGGAACTTCTCTCTGTGTCTGTCT	GGACTAGTTCCAGCATGATTCCTGTGGTATCTT
Primers used for real-time PCR		
Name	Forward primers (5’-3’)	Reverse primers (5’-3’)
Actin	ATCCGGAATCAAACGCACCCT	AGAGCGTCAGCGAAGAAGGAAG
ATP synthase	CGATTTCCGCATCCGCATCGAG	GAGCCCTTCCCGGTGAACTCAT
YL	TACCCTTCCTTCAATCCTTCCAC	TGAGCATCTTTCTCACTGCCAAG

Primers used for verifying RNA editing sites by Sanger sequencing		
Name	Forward primers (5'-3')	Reverse primers (5'-3')
accD	ATGGAAAAATGGTGGTTTAATTCTAT	CTATGCCTTATCATTTTTAGTCAAAG
atpF	ATGAAAAATATAACCGATTCTTTCC	CTAATCAGTTATTTTTTCTTTCATCGTC
clpP	TTATTCAACGGCTACAAGATCTACA	ATGCCCATTGGTGTTCCAAGAGTAC
ndhA	ATGATAATTTATTTAACAGAGATAC	TTACAGTGAAAACAGTTGAGACGAA
ndhB	ATGATCTGGCATGTACAGAATGAAA	CTAAAAAAGGGTATCCTGAGCAATT
ndhC	ATGTTTCTTCTTTACGAATATGATA	TTAAGACCATTCTAATGCCCCCTTT
ndhD	ACGAATTATTTTCCTTGGTTAACAACAG	CTATAAAAAATTAGATAGAATAGCTTCA
petB	ATGAGTGTGCGGCTTGTTTTAATGGATC	CTATAAAGGACCAGAAATGCCTTGCTTA
psaI	ATGATAAACTTTCCTTCCATTT	CTAAAAAATCTTGTTTTTTTGAAC
psbE	CTAAAAAGATCTACTAAATTCATCAAGT	ATGTCTGGAAGCACAGGAGAACGTT
rpl23	ATGAATGGAATAAAATATGCAGTAT	TTAAGTTTTTTTCTTTCTAAGAGGT
rpoA	TTATGCAAAATGCTTTTCTATTT	ATGGTTCGAGAGAAAATAAGAGT
rpoB	ATGCTTGGGGATGGAAATGAAG	TTAAGCTTCCTTCCTATGAATCCGG
rpoC1	ATGATTGATCAGTATAAACATCAACAAC	TTAGATATCATATGAATAGGCCCGG
rps2	ATGACAAAAAGATATTGGAACATAA	TCAAGAATTTCGTATATAGCTAGAA
rps14	ATGGCAAGGAAAAGTGTGATTCAGA	TTACCAACTGGATCTTGTTGCACCG
rps16	ATGGTAAAACTTCGTTTGAAACGAT	TTAATGGAATTTCGTTTGTTGATTC
rps18	ATGGAAAAATCTAAGCGACTCTTTATGA	TTAATTTCTATATTTTTTTTTTCTTAAA
